# Iodine-124: A Promising Positron Emitter for Organic PET Chemistry

**DOI:** 10.3390/molecules15042686

**Published:** 2010-04-13

**Authors:** Lena Koehler, Katherine Gagnon, Steve McQuarrie, Frank Wuest

**Affiliations:** 1 Institute of Radiopharmacy, Research Center Dresden-Rossendorf, Dresden, Germany; E-Mail: lenakoehler@googlemail.com (L.K.); 2 Department of Physics, University of Alberta, Edmonton, Canada; E-Mail: kgagnon@phys.ualberta.ca (K.G.); 3 Department of Oncology, University of Alberta, Edmonton, Canada; E-Mail: steve.Mcquarrie@albertahealthservices.ca (S.M.)

**Keywords:** iodine-124, positron emission tomography (PET), molecular imaging

## Abstract

The use of radiopharmaceuticals for molecular imaging of biochemical and physiological processes *in vivo* has evolved into an important diagnostic tool in modern nuclear medicine and medical research. Positron emission tomography (PET) is currently the most sophisticated molecular imaging methodology, mainly due to the unrivalled high sensitivity which allows for the studying of biochemistry *in vivo* on the molecular level. The most frequently used radionuclides for PET have relatively short half-lives (e.g. ^11^C: 20.4 min; ^18^F: 109.8 min) which may limit both the synthesis procedures and the time frame of PET studies. Iodine-124 (^124^I, t_1/2 _= 4.2 d) is an alternative long-lived PET radionuclide attracting increasing interest for long term clinical and small animal PET studies. The present review gives a survey on the use of ^124^I as promising PET radionuclide for molecular imaging. The first part describes the production of ^124^I. The second part covers basic radiochemistry with ^124^I focused on the synthesis of ^124^I-labeled compounds for molecular imaging purposes. The review concludes with a summary and an outlook on the future prospective of using the long-lived positron emitter ^124^I in the field of organic PET chemistry and molecular imaging.

## 1. Introduction

The convergence of molecular and cellular biology with imaging sciences to molecular imaging has revolutionized current biomedical research. Molecular imaging is defined as the *in vivo* characterization and measurement of biologic processes at the cellular and molecular level [1,2,3]. Molecular imaging aims at developing non-invasive strategies for characterizing the molecular and metabolic profiling in living subjects. Molecular and cellular processes can be studied and visualized at various levels of resolution by means of *in vivo* imaging techniques, which span from ultrasonic to gamma-ray frequencies. In recent years, positron emission tomography (PET) has become a powerful non-invasive molecular imaging technique which provides functional information of physiological, biochemical and pharmacological processes in laboratory animals and humans [4,5,6]. The possibility to observe molecular interactions in living organisms and to determine absolute values of physiological parameters places PET in a unique position among other molecular imaging techniques. In a typical PET study the PET radiotracer, a compound labeled with a short-lived positron emitter, is injected intravenously into a human or animal. Tissue concentrations of the radiotracer are measured over time, and these data are combined with information on plasma probe concentration of the radiotracer to assay metabolism. Mathematical methods for the evaluation of PET measurements within the framework of compartment models are well established [7,8]. Within the spectrum of available positron emitters, fluorine-18 (^18^F) is an almost ideal radionuclide for PET, thanks to its ease of production and favorable physical properties, such as a 109.8 min half-life and low β^+^ energy (0.64 MeV). The success of various ^18^F-labeled radiotracers like 2-[^18^F]fluoro-2-deoxy-D-glucose ([^18^F]-FDG) as metabolic markers in biomedical research and clinical practice has prompted research on the potential of other positron-emitting radionuclides with longer half-lives. The choice of the appropriate radionuclide is among the most important aspects for the design and application of novel PET radiotracers. The physical half-life of the radionuclide should reflect the timeframe of the biological process to be studies. Various excellent reviews have addressed and discussed the importance of other positron-emitting radionuclides in the design of novel radiopharmaceuticals [9,10,11].

Several other positron emitting radionuclides with different physical half-lives can be prepared in high yields by means of small biomedical cyclotrons. Prominent examples of positron-emitting radionuclides with longer half-lives include copper-64 (^64^Cu, t_1/2_ = 12.7 h), yttrium-86 (^86^Y, t_1/2_ = 14.7 h), bromine-76 (^76^Br, t_1/2_ = 16.2 h), and iodine-124 (^124^I, t_1/2_ = 4.2 d).

In recent years, the positron emitting halogen ^124^I has become an attractive long-lived radionuclide for the design and synthesis of novel PET radiotracers. Its convenient 4.2 d half-life allows extended radiosynthesis protocols and longitudinal PET imaging studies. Moreover, labeling chemistry for ^124^I is well established, and a wide variety of compounds have been labeled for molecular imaging purposes with PET. 

The present review gives a survey on the use of ^124^I as promising PET radionuclide for molecular imaging. The first part of the review deals with the production, processing and PET imaging of ^124^I. The second part covers basic radiochemistry with ^124^I focused on the synthesis of ^124^I-labeled compounds for molecular imaging purposes. The review concludes with a summary and an outlook on the future prospective of using the long-lived positron emitter ^124^I in the field of organic PET chemistry and molecular imaging.

## 2. Production, processing, and PET imaging of ^124^I

### 2.1. ^124^I production routes

Early investigations into the production of ^124^I most commonly employed the ^124^Te(d,2n)^124^I nuclear reaction scheme [12,13,14,15,16]. More recently however, with the increase in the number of low-energy proton cyclotrons (for the purpose of producing traditional PET isotopes such as ^18^F or ^11^C), the ^124^Te(p,n)^124^I reaction has been gaining popularity [15,17,18,19,20,21]. Despite the slight decrease in yields noted with the ^124^Te(p,n)^124^I nuclear reaction (Table 1), this scheme offers the possibility of obtaining the highest levels of ^124^I radioiodine purity at the time of administration. 

**Table 1:** Selection of published data on ^124^I production.

Nuclear reaction	Effective Energy [MeV]	Targetmaterial	Enrichment [%]	Yield [MBq/μAh]	Radioiodine impurities [%]	Reference
^124^Te(p,n)^124^I	13→9	Te	99.51	20^ a^	^123^I(41)	[17]
	12.2→0	TeO_2_	99.8	13	^123^I(10.039), ^125^I(0.018), ^126^I(0.041), ^130^I(0.379)	[15]
	13.5→9	TeO_2 _/ 5% Al_2_O_3_	99.8	5.8	^125^I(0.01), ^126^I(<0.0001)	[18]
	12.5→5	TeO_2_	99.3	9.0 ± 1.0	^125^I(0.053 ± 0.015)	[19]
	11→2.5	TeO_2 _/ 6% Al_2_O_3_	99.5	6.40 ± 0.44	^125^I(<0.02), ^126^I(<0.001)	[20]
	14→7	TeO_2 _/ 5% Al_2_O_3_	99.86	21.1	^125^I(0.03), ^126^I(0.007)	[21]
^125^Te(p,2n)^124^I	20.1→10.5	TeO_2_	93	43.3	^123^I(8), ^125^I(5)	[25]
	22→4	Te	98.3	111^a^	^125^I(0.89)	[26]
	21→15	Te	98.3	81^ a^	^123^I(7.4), ^125^I(0.9)	[24]
	22	TeO_2_	98.5	104	^123^I(<1)	[27]
^126^Te(p,3n)^124^I	36.8→33.6	Te	Nat	67 ^a^	--	[28]
	38→28	Te	> 98	148^ a^	^123^I(84), ^125^I(1.5), ^126^I(1.4)	[29]
^123^Te(d,n)^124^I	11→6	Te	91.0, 85.4	2.8^ a^	^123^I(3321 ^b^)	[30]
^124^Te(d,2n)^124^I	15→0	Te	95	20.4 ± 2.2	^126^I(0.5)	[12]
	15→8	Te	91.7	18.9	^125^I(0.35 ^b^), ^126^I(0.39 ^b^), ^131^I(0.08 ^b^)	[13]
	16→6	Te	96.7	23.7^ a^	--	[14]
	14→0	TeO_2_	89.6	15	^123^I(1.16), ^125^I(1.41), ^126^I(1.16), ^130^I (7.87), ^131^I(0.31)	[15]
	14→10	Te	99.8	17.5^ a^	^125^I(1.7)	[16]
^nat^Sb(α,xn)^124^I	22→13	Sb	Nat	1.02^ a^	^123^I(892 ^b^), ^125^I(13 ^b^), ^126^I(0.16 ^b^)	[31]
^121^Sb(α,n)^124^I	22→13	Sb	99.45	2.11^ a^	^123^I(891 ^b^), ^125^I(<0.2), ^126^I(<0.2)	[31]
^nat^Sb(^3^He,xn)^124^I	35→13	Sb	Nat	0.95^ a^	^121^I(37700 ^b^), ^123^I(3877 ^b^), ^125^I(0.6), ^126^I(0.6)	[32]

^a^ Based on experimental cross section data; ^b^ Percent calculated here from the ratio of the published saturation yield data.

Prior to examining the difference between the ^124^I radioiodine purity of these two reactions, it is important to note that radioiodine contaminants may arise from one of two sources [22]. Firstly, as the natural composition of tellurium is given as ^120^Te (0.09%), ^122^Te (2.55%), ^123^Te (0.89%), ^124^Te (4.74%), ^125^Te (7.07%), ^126^Te (18.84%), ^128^Te (31.74%), and ^130^Te (34.08%) [23], the ^124^Te target material must be highly enriched as the presence of other tellurium isotopes will permit reactions which give rise to radioiodine contaminants. This source of contaminants is not however the limiting concern when producing ^124^I as target materials of higher enrichment may always be employed. Although enrichment levels greater than 99 percent are common (Table 1), the high cost of the enriched tellurium imposes the need for recycling the irradiated target material [13]. 

The second source of radioiodine contaminants arises from the fact that, despite 100 percent enrichment, competing reactions may occur during the ^124^Te irradiation. For the energy range employed in the ^124^Te(p,n)^124^I and ^124^Te(d,2n)^124^I reactions, the primary competing reactions of concern are ^124^Te(p,2n)^123^I and ^124^Te(d,n)^125^I, respectively. Given the difference in half-lives for ^123^I (t ½ = 13.2 hrs) and ^125^I (t ½ = 59.4 days), a mixture containing ^123^I and ^124^I will present a greater ^124^I purity over time. In contrast, a mixture of ^124^I and ^125^I will present a decrease in ^124^I purity with time. It is for this reason that although the ^124^Te(p,n)^124^I may present with a lower ^124^I radioiodine purity than ^124^Te(d,2n)^124^I at the end of the irradiation, the ^124^Te(p,n)^124^I reaction will give rise to a higher radioiodine purity following a period of decay [15]. 

As the probability for competing reactions to occur during the irradiation of a particular tellurium isotope depends upon the irradiation energy, it is common to strategically select the irradiation energy window to maximize the production of ^124^I while minimizing the radioiodine impurities [14,16,24]. The selection of this energy window typically results in a trade-off between purity and yield. For example, although the ^123^I contaminant arising from the ^124^Te(p,2n)^123^I reaction may be minimized by reducing the incident proton energy, a decrease in energy from 13 MeV to 11 MeV results in a near three-fold decrease in the ^124^I yield [20]. In some cases, for example the ^126^Te(p,3n)^124^I reaction, if the entire proton energy were to be deposited in the ^126^Te target, large ^125^I and ^126^I impurities from the competing ^126^Te(p,2n)^125^I and ^126^Te(p,n)^126^I reactions would result. To minimize these impurities, the exit energy is controlled by varying the thickness of the target material. 

In examining the three proton induced reactions on enriched tellurium, the ^125^Te(p,2n)^124^I [24,25,26,27] and ^126^Te(p,3n)^124^I [28,29] reaction pathways offer 5–9 times higher ^124^I yields than the ^124^Te(p,n)^124^I strategy [29]. The major shortcomings to these two reaction schemes are that, (i) higher proton energies are needed and, (ii) the level of long-lived radioiodine contaminants is increased. A search for alternative methods of producing ^124^I has lead to investigations of the ^123^Te(d,n)^124^I [30], ^nat^Sb(α,xn)^124^I [13,31], ^121^Sb(α,n)^124^I [31], and ^nat^Sb(^3^He,xn)^124^I [32] reactions. As a result of the low yields reported, these four reaction schemes are not ideal for clinical production of ^124^I. Given the low yields observed for the antimony-based reactions, the remainder of the discussion on ^124^I production and recovery is limited to tellurium-based target materials. 

The number of reaction strategies available for the production ^124^I at a particular facility is dictated by the irradiation energies and particles available. If multiple schemes are possible, the choice of which reaction strategy to employ requires a thorough evaluation of the desired ^124^I yields and the tolerable level of radioiodine impurities at the time of administration. As noted by Scholten *et al.* [29], although ^124^I of the highest radioiodine purity may be recommended for diagnostic applications, a higher impurity level may perhaps be more tolerable when harnessed for therapeutic purposes. 

### 2.2. Thermal design and irradiation considerations

As the total ^124^I activity produced is proportional to the current at which the target is irradiated, higher currents are always desired (particularly given the generally low yield of ^124^I). Unfortunately, due to the large power dissipated into the target material (e.g. 300 W given 20 μA of 15 MeV protons) the maximum current at which a target may be irradiated is dictated by the thermal performance of the target material itself. Evidence of inadequate thermal performance during the irradiation is given by the presence of volatile ^124^I and/or tellurium [33,34], or in some cases, significant melting of the expensive tellurium target material [35,36]. 

To maintain a superior thermal performance during high-current target irradiations, extensive cooling of the target material and support plate is essential. Commonly, this is achieved through water cooling on the back of the target support plate and helium cooling on the front surface of the tellurium target material [18,21,37,38]. Although 4 π water cooling has been employed [18,26,27], Qaim *et al.* [18,26] have noted appreciable losses of ^124^I to the cooling water with this configuration. For the purpose of improving the target cooling efficiency, several studies have examined the use of finite-method based computer simulations to model the heat transport during the target irradiation [34,39,40,41].

The chemical and physical form of the tellurium has an impact on the thermal stability of the irradiated target. The most common target material employed for ^124^I production is TeO_2_ (m.p. 733 ºC). This material is attributed to having better thermal characteristics when compared to tellurium metal [33,37]. It is common to add 5–7% by mass Al_2_O_3_ to the TeO_2_[18,20,21,37,42,43] for the purpose of (i) enhancing the binding of the TeO_2_ to the target plate [18,37,43,44], (ii) giving the target material a glassy solid structure thus eliminating the need for a cover foil [37,44], and (iii) increasing the uniformity of the target material layer [44]. As investigated by Nye *et al.* [33], Al_2_Te_3_ appears to be a promising target material given its high melting point (m.p. 895 ºC), high tellurium mass fraction and its formation of a glassy-melt. McCarthy *et* al. [45], and Rowland *et al.* [46] have also investigated the use of Cu_2_Te (m.p. 1132 ºC). For the purpose of improving the thermal performance of elemental tellurium targets, Sadeghi *et al.* [47] and Yanbawi *et al.* [48] have recently explored methods for optimizing tellurium electrodeposition techniques.

In addition to the tellurium target material, the support plate in which the target material is deposited must be considered. In selecting a support plate, Bosch *et al.* [49] discuss how its selection is a compromise between thermal conductivity, chemical resistance, adhesion of the tellurium target material and desire for low and short-lived, non-volatile, induced radioactivity at temperatures ranging from room temperature to the melting point of the target material. Although platinum generally serves this purpose (commonly with furrows scratched into the surface [21,37], or platinum gauze employed [18,49] to increase the adhesion with the target material), both Sheh *et al.* [37] and Glaser *et al.* [19] report a warping of the platinum support plate. While Sheh *et al.* [37] attribute this deformation to a misalignment of the proton beam, Glaser *et al.* [19] suggest that the warping occurs as a result of the differences in the thermal expansion coefficients between TeO_2_ and platinum. As an alternative support plate, Alekseev *et al. *[50] suggest that tantalum offers a good compromise between thermal conductivity and strength. They claim however that it is impossible to form a strong elemental tellurium coating directly onto the tantalum. To overcome this challenge, they electroplate nickel onto the tantalum to act as an intermediate binding layer. Examples of other target plate support materials which have been employed for ^124^I production include nickel electroplated copper [12,47], tungsten [45], platinum coated tungsten [46], silicon [45], platinum (90%) iridium (10 %) [15,51,52], and rhodium electroplated stainless steel [53].

In addition to target cooling and material selection, there are three more factors which are also commonly varied for the purpose of increasing the thermal performance of the irradiated target. First, as was discussed in the previous section, the target thickness is often reduced so that the entire beam energy is not deposited within the target itself. This reduction in thickness not only serves the purpose of limiting the competing reactions which may give rise to radioiodine impurities, it allows for some of the heat to be deposited into the target support plate. The reduction in thickness also offers the added benefit of reducing the material costs [24]. Second, the power density to the target is commonly reduced by slanting the irradiated target [20,21,27,37,51]. This incline increases both the area over which the heat is deposited and the effective target thickness. Finally, to achieve a more uniform heat distribution, and avoid potential “hot-spots”, it is common to defocus or wobble/sweep the beam across the target [14,18,21,38,42].

Although the thermal performance of a target is often considered to be the current-limiting factor, several novel studies have investigated methods for exploiting the ^124^I volatility during irradiation for the purpose of performing on-line ^124^I extraction. Examples of such studies include the Te(70)/Tl(30) eutectic (m.p. 224 ºC) investigated by Zyuzin *et al.* and Johnson *et al.* [54,55], the TeO_2_-based target system (m.p. 733 ºC) explored by Stevenson *et al.* [53], and the tellurium metal-based system (m.p. 452 ºC) evaluated by Runz *et al.* [36]. 

### 2.3. Processing: Dry distillation of the ^124^I

Of the two methods employed for separating ^124^I from an irradiated tellurium target, dry distillation is commonly preferred over wet chemical processing as it is reasonably straightforward to perform and allows for recycling of the target without further handling [51]. As dry distillation requires a material with good melting and solidification properties, TeO_2_ is most commonly employed since elemental tellurium has a tendency to “blow up” upon heating [49]. A conventional dry distillation setup entails the heating of an irradiated target under gas flow in a quartz tube. Given the increased vapor pressure of the iodine compared to the tellurium target material, the iodine is transported through the quartz tube and trapped downstream of the furnace. Although the following summary focuses on the dry distillation of TeO_2_, successful dry distillation ofAl_2_Te_3_ [33] and Cu_2_Te [45,46] have been reported. 

For the purpose of maximizing the ^124^I recovery while minimizing the loss of the expensive tellurium target material, a wide variation in the setup parameters is noted in the literature. While the distillation time is commonly kept relatively short (typically varied from 5–20 min [18,19,37,51]), the distillation temperatures reported in the literature show a wide variation 670 ºC [37]–820 ºC [25]. It should be noted that this temperature variation may perhaps be attributed, in part, to differences between the oven temperature set-point and the true target material temperature (a 40 ºC difference in these two values was noted by Sheh *et al.* [37]). As the tellurium loss has been related to increased distillation times and increased temperatures, both Qaim *et al.* [18] and Scholten *et al.* [29] recommend that a 15 min distillation at 750 ºC offers a good compromise between the tellurium loss and ^124^I recovery. In contrast, Zweit *et al.* [28] report optimal distillation conditions of 770 ± 5 ºC for 20 min. To further reduce the heating time required for release of the ^124^I, the use of an induction furnace, as opposed to the conventional tube oven has been proposed [45,46,56]. 

As noted by Glaser *et al.* [19] the tellurium detected in the final ^124^I product vial increases as a target undergoes repeated irradiation/processing cycles. To minimize the metallic tellurium from reaching the ^124^I trap, the use of an Al_2_O_3_/quartz wool filter has been reported [19,57]. Qaim *et al.* [18] place a strong emphasis on the need for annealing the TeO_2_/Al_2_O_3_ target mixture at 450 ºC for the purpose of converting small amounts of TeO_3_ to TeO_2_. If this step is not performed, they report an increase in tellurium loss during the distillation process. Although air [15,18,21,29], argon [43], helium [53] and oxygen [19,27,28,37,38] have all been employed as carrier gases during the ^124^I dry distillation process, Glaser *et al.* [19] note the preference of an oxygen atmosphere for the purpose of regenerating the target as reduced tellurium has a higher volatility than TeO_2_. Carrier gas flow rates reported in the literature typically range from 15–40 mL/min [20,21,25,43,51], however, rates as low as 5 mL/min [37] and as high as 80 mL/min [27] have been reported. Although there is limited discussion in the literature as to flow rate optimization, Zweit *et al.* [28] report an optimal oxygen flow rate of 24 mL/min. 

The trap employed for collecting the ^124^I typically takes one of two forms. Firstly, the trap may be a solution of NaOH. Typical NaOH volumes range from 100–1000 μL [18,21,38], while typical concentrations range from 0.001–0.1 M [21,25,38]. The second trap design involves the use of a capillary tube. Reported tube materials include stainless steel [15,51], Pyrex [37] and quartz [20,43]. To increase the trapping efficiency, the capillary tubes may be primed with NaOH [37,51]. Loading of the capillary tube with platinum wire has also been reported for the purpose of increasing the surface area [20]. The ^124^I which has settled onto the surface of the capillary is recovered by washing the capillary with a weak buffer solution [20,37]. To enhance the ^124^I trapping, the NaOH or capillary traps may be cooled [20,21,37]. To prevent premature precipitation of the distilled iodine in the region between the furnace and the trap, this region is commonly heated using a stream of hot air [18,29] or a heat wire/ribbon [19,20,27,49]. A temperature of 200 ºC is recommended in this region for ensuring efficient ^124^I transport [19,49]. For the purpose of preventing the release of any un-trapped volatile ^124^I, a second NaOH trap [18,27,29], or a charcoal filter [19,49] is often placed downstream of the primary ^124^I trap. 

### 2.4. PET imaging of ^124^I

PET relies on the detection of the two back-to-back 511 keV photons in coincidence. These two photons arise from positron annihilation following the positron (β^+^) decay of the radioisotope on the labelled pharmaceutical. As these two annihilation photons are emitted in opposite directions (nearly 180º), the annihilation event is taken to have occurred along the path joining the two detectors (referred to as the line of response (LOR)). It is the acquisition of many such LORs which allows for localization of the radiopharmaceutical uptake within a patient. PET imaging of ^124^I introduces several challenges over the imaging of traditional PET isotopes (*i.e.,*^18^F and ^11^C). 

Firstly, for the traditional PET isotopes, β^+^ decay occurs greater than 96 percent of the time. For ^124^I however, β^+^ decay occurs only 23 percent of the time with the positrons having higher energies when compared with the traditional PET isotopes. This low branching ratio results in the need for longer imaging times to achieve similar counting statistics assuming similar starting activities [58]. 

Next, the traditional PET isotopes always decay to the ground state of the daughter nucleus. For ^124^I however, the decay scheme is significantly more complex. Approximately 65 percent of the time, the decay of ^124^I populates an excited state of ^124^Te. The consequence of decaying to an excited state (as opposed to the ground state) is that a cascade of high energy photons follows the ^124^I decay as the ^124^Te nucleus de-excites. As there is no angular correlation between the annihilation photons and the cascade photons [59,60], the detection of a cascade photon and an annihilation photon within the timing window of the PET scanner will give rise to a falsely identified LOR. If uncorrected, these prompt gamma coincidences (PGC) will lead to spurious activity in the final reconstructed image [59]. To account for the PGCs in non-conventional PET radionuclides, it is common to encompass corrections within the image reconstruction process [59,61,62,63] and/or optimize the image acquisition parameters to limit the detection of the cascade photons [60,64,65]. 

It should also be noted that as a result of these additional high energy cascade photons, the high energy positrons associated with ^124^I decay, and the long half-life of ^124^I, the radiation burden associated with ^124^I is increased when compared with the traditional PET isotopes. This results in lower injected activities of ^124^I-labelled radiopharmaceuticals, and in turn, the need for increased imaging times to achieve the desired counting statistics. 

Finally, as a result of the increased positron energy when compared to the positrons arising from the decay of the traditional PET isotopes, the positrons will travel a greater distance prior to annihilation. Although this increased distance results in a degradation of the image point spread function [66], Liu and Laforest [67] have recently reported a method for accurately determining the activity concentration in small lesions for PET imaging of several long range position emitters. 

## 3. Basic Principles of Radiochemistry with Radioiodine Isotopes

Radioiodination chemistry generally employs the same chemistry used in organic synthetic chemistry involving non-radioactive iodine. However, the synthetic methods involving radioiodine nuclides must be adapted to special reaction conditions considering the half-life of the radionuclide and the use of small-scale concentrations. In recent decades, methods of radioiodination have been reviewed extensively in the literature with emphasis placed on labeling methods, mechanistic aspects, and applications of radioiodinated compounds [68,69].

Radiochemistry involving radioiodine nuclides can principally be subdivided into nucleophilic and electrophilic substitution reactions as the most general synthesis routes. These two reaction schemes are discussed in this review. A special discussion is also included on the radioiodine labeling of macromolecules such as peptides, proteins and antibodies.

The ease of oxidizing iodide into an electrophilic form of iodine makes electrophilic labeling the most frequently and most popular employed radioiodination method. The advantage of the easy oxidation of iodide in comparison with other radiohalogens (except astatine) and the subsequent straightforward electrophilic labeling chemistry is somewhat compromised by the lower stability of the carbon-iodine bond. The low stability of the carbon-iodine bond may result in substantial radiodeiodination *in vivo*, leading to the formation of radioiodide and its subsequent rapid accumulation in the thyroid and stomach. Aromatic carbon-iodine bonds are more stable than aliphatic carbon-iodine bonds. As a consequence, compounds containing an aromatic or vinylic carbon-iodine bond are more often prepared to confer as high metabolic stability towards radiodeiodination *in vivo* as possible.

### 3.1. Nucleophilic substitution reactions

Halogen exchange reactions are the most common method for the introduction of radioiodine into organic molecules. The nucleophilic exchange reactions can occur in aliphatic and aromatic compounds according to the nature of the leaving group. Compared to nucleophilic substitutions with aliphatic compounds, nucleophilic substitution reactions proceed slowly on aromatic compounds. Moreover, the aromatic substrate must be activated by electron-withdrawing groups. To accelerate the exchange reactions in aromatic compounds, various metal-assisted reactions proved to be quite successful for nucleophilic radioiodinations of arenes. Prominent examples include the use of ammonium salts or copper(I)- and copper(II)-assisted radioiodinations. Other methods involve treatment of aromatic diazonium salts or decomposition of triazenes in the presence of protic solvents or Lewis acids with sodium radioiodide.

### 3.2. Electrophilic substitution reactions

Electrophilic radioiodination reactions are the preferred method for radioiodinations of aromatic compounds. The method relies on the ease with which radioiodide can be oxidized *in situ* to a positively charged iodine (I^+^) species by means of various mild oxidizing agents. Commonly employed oxidizing agents include iodine monohalides like ICl, *N*-chloroamides like chloramine T, peracids like peracetic acid, *N*-halosuccinimides like *N*-chlorosuccinimide, and other oxidants like metal ions (e.g. Tl^3+^, Ce^4+^), iodate and enzymes (e.g. lactoperoxidase).

Electrophilic radioiodinations on an aromatic system can be performed directly (radioiodo-deprotonation) or by means of various demetallation techniques. Radioiodo-demetallation reactions require organometallic compounds as precursors. Radio-demetallation reactions with electropositive radioiodine involving organometallic compounds like organoboranes, group IV metal-containing compounds (Si, Ge, Sn), and organomercury and organothallium compounds usually proceed in high radiochemical yield in a highly regioselective manner.

Among the known demetallation reactions, destannylation reactions are the most favoured method for electrophilic radioiodinations. Radioiodo-destannylation reactions proceed well under fairly mild reaction conditions affording the radioiodinated product in high radiochemical yields and high regioselectivity even by using only very small quantities of organotin precursors (<50 μg). 

### 3.3. Radioiodination of peptides and proteins

Various methods for the radioiodination of proteins and antibodies under mild reaction conditions compatible with the structural and functional integrity of proteins have been developed. Methods include the direct labeling of proteins through radioiodination of tyrosine residues with electropositive radioiodine. Chloramine T, Iodogen^®^, and various oxidative enzymes are useful oxidizing agents for the mild *in situ* oxidation of radioiodide for direct protein labeling. 

Another approach deals with the application of radioiodinated bifunctional labeling precursors, also referred to as prosthetic groups. Here the peptide or protein is reacted with an iodinated molecule which has been activated for conjugation with functional groups of the peptide or protein. Commonly used functional groups of peptides or proteins for conjugation with prosthetic groups are amine and sulfhydryl groups as typically found in lysine and cysteine residues.

## 4. Examples for ^124^I-labeled Compounds

The following part of the review summarizes the synthesis and application of various ^124^I-labeled compounds as molecular imaging probes for PET. The survey is organized based on the employed synthesis route for the preparation of the ^124^I-labed compounds. 

### 4.1. ^124^I-labeled compounds prepared via nucleophilic exchange reactions

#### 4.1.1. meta-[^124^I]Iodobenzylguanidine ([^124^I]MIBG)

**Scheme 1:**
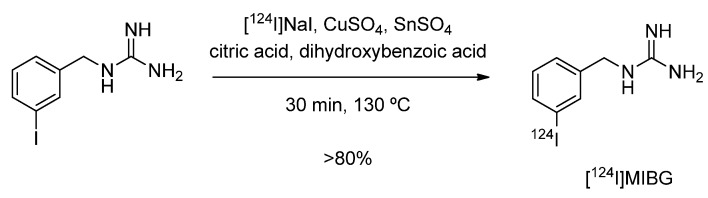
[^124^I]MIBG synthesis via Cu(I)-assisted nucleophilic exchange reaction.

*m*-Iodobenzylguanidine (MIBG) is used in diagnosis (labeled with ^123^I or ^124^I) and therapy (labeled with ^131^I) of neuroblastoma and pheochromacytoma. A recent study describes the use of ^124^I-labeled MIBG for imaging norepinephrine transporter (NET) function [70]. The synthesis of [^124^I]MIBG was performed according to a nucleophilic halogen exchange reaction with [^124^I]NaI in the presence of Cu(I) at elevated temperatures. Copper(I) was generated *in situ* through reduction of Cu(II)-sulfate with tin(II). The overall radiochemical yield was greater than 80%. The reaction is given in Scheme 1.

#### 4.1.2. 1-α-D-(5-deoxy-5-[^124^I]iodo-arabinofuranosyl)-2-nitroimidazole ([^124^I]IAZA)

**Scheme 2:**
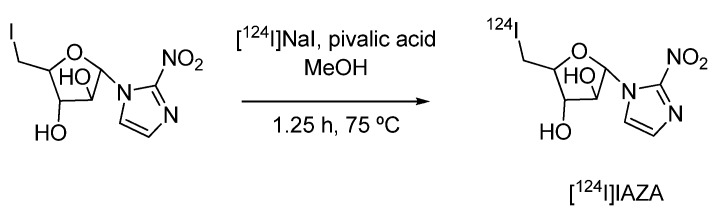
Synthesis of hypoxia imaging agent [^124^I]IAZA.

The hypoxia imaging agent [^124^I]IAZA was used in a comparative study with two other 2-nitro-imidazole derivatives ([^18^F]FMISO and [^18^F]FAZA) for the visualization of tumor hypoxia in A431 bearing mice by means of PET [71]. [^124^I]IAZA was prepared by an isotopic exchange reaction with [^124^I]NaI (Scheme 2).

#### 4.1.3. [^124^I]Iodo-azomycin-galactopyranoside ([^124^I]IAZG)

**Scheme 3:**
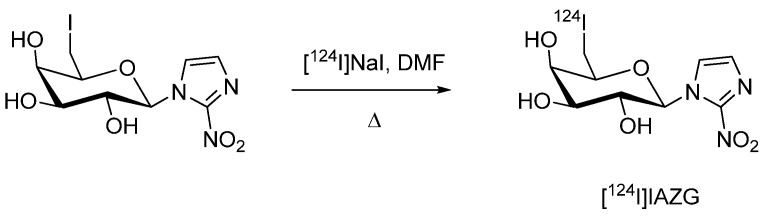
Synthesis of hypoxia imaging agent [^124^I]IAZG.

[^124^I]Iodo-azomycin-galactopyranoside ([^124^I]IAZG) as another ^124^I-labeled hypoxia imaging agent was reported by Zanzonico *et al. *[72]. [^124^I]IAZG was prepared based on a nucleophilic isotope exchange reaction according to a literature procedure involving iodine-125 and iodine-123 (Scheme 3) [73,74,75]. 

Small animal PET studies with [^124^I]IAZG and [^18^F]FMISO, another hypoxia PET tracer, were performed in MCa breast tumors and FSaII fibrosarcomas implanted in mice. [^124^I]IAZG showed high uptake in large tumors. PET imaging with [^124^I]IAZG was optimal after 24 h post injection (p.i.), when the whole body background activity had dissipated. As a result, [^124^I]IAZG showed a tumor uptake of 17% at 48 h p.i., whereas tumor uptake for [^18^F]FMISO was 5–10% at 3–6 h p.i..

Comparable results were observed for Morris hepatoma McA-R-7777 tumors as reported by Riedl *et al.* [74,75]. Substantial uptake of radioactivity in the thyroid accounting for 30% of the total-body activity at 48 h p.i. is indicative of radiodeiodination of [^124^I]IAZG *in vivo*. The presented data indicate the potential of [^124^I]IAZG for hypoxia imaging.

#### 4.1.4. 1-(2-Deoxy--β-D-ribofuranosyl)-2,4-difluoro-5-[^124^I]iodobenzene ([^124^I]dRFIB)

**Scheme 4:**
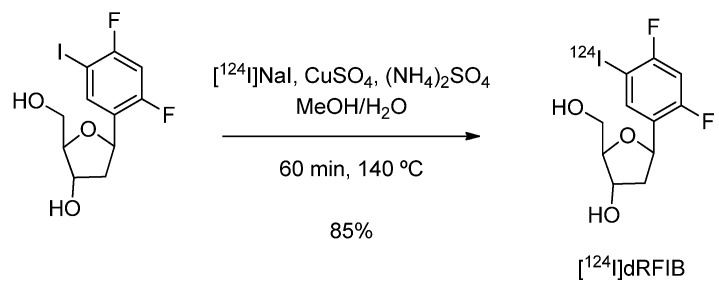
Synthesis of [^124^I]dRFIB.

1-(2-Deoxy-β-D-ribofuranosyl)-2,4-difluoro-5-[^124^I]iodobenzene ([^124^I]dRFIB) for imaging cell proliferation was introduced by Stahlschmidt *et al. *[76]. The radiotracer was synthesised in five steps following the literature-reported method and radioiodination was accomplished via nucleophilic isotope exchange reaction in the presence of copper sulphate and ammonium sulphate (Scheme 4). 

The precursor was mixed with a solution containing copper and ammonium sulphate in water. The required amount of [^124^I]NaI was added in NaOH. The vial was sealed, and a venting needle attached to an activated carbon cartridge was used. The solvent was evaporated in a stream of dry nitrogen and the residue was heated. The product was purified and isolated by means of a C_18_ cartridge and HPLC. The reaction was optimized through screening different temperatures and precursor amounts. A radiochemical yield of 2.4% was obtained at 60 ºC, which was increased to 10% at 80 ºC and 76% at 100 ºC. Optimized reaction conditions provided [^124^I]dRFIB in radiochemical yields of up to 85% within one hour. No data on the radiopharmacological evaluation of [^124^I]dRFIB are reported.

### 4.2. ^124^I-labeled compounds prepared via electrophilic substitution reactions

#### 4.2.1. Direct electrophilic substitution on activated aromatic systems

#### 4.2.1.1. 5-[^124^I]Iodo-2′-deoxyuridine ([^124^I]IUdR)

**Scheme 5:**
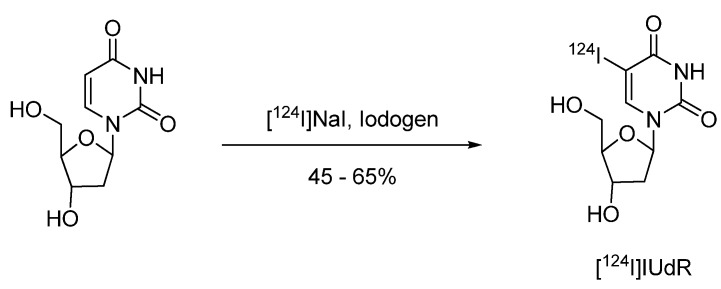
Synthesis of 5-[^124^I]iodo-2’-deoxyuridine ([^124^I]IUdR).

Radiosynthesis of 5-[^124^I]Iiodo-2′-deoxyuridine ([^124^I]IUdR) for functional imaging of cell proliferation by means of PET was investigated by Guenther *et al.* [77]. Radiolabeling of [^124^I]IUdR was performed via direct electrophilic substitution method using Iodogen^®^ as the oxidising agent (Scheme 5). 

2'-Deoxyuridine as the labeling precursor was dissolved in 0.2 M phosphate buffer (250 µL) and added to an Iodogen^®^-coated Reacti-vial. After the addition of 185 MBq of [^124^I]NaI, the reaction was performed at 65 ºC for 15 min. After cooling to room temperature the product was separated via solid phase extraction on a Sep-Pak C-18 cartridge. The cartridge was eluted with ethanol which was evaporated under a stream of nitrogen. The residue was re-dissolved in saline and filtered through a 0.22 µm sterile filter. The labeling yield was 45–65% and the purity was greater 93% [77]. [^124^I]IUdR showed rapid radiodeiodination *in vivo* resulting in high accumulation of activity in the thyroid. After 24 h p.i. brain tumor imaging with [^124^I]IUdR was feasible [78].

#### 4.2.1.2. ^124^I-labeled hypericin

Recently, Kim *et al.* described the synthesis of ^123^I- and ^124^I-labeled hypericin derivatives [79]. Hypericin, a natural polycyclic aromatic dianthraquinon, was used in treatment of depression and showed antiretroviral activity against several viruses including human immunodeficiency virus (HIV). Additionally, an elevated activity against protein kinase C (PK-C) was found in malignant gliomas. Kim *et al.* investigated the possibility of using iodine labeled hypericin derivatives for imaging malignant gliomas with PET and SPECT [79].

**Scheme 6:**
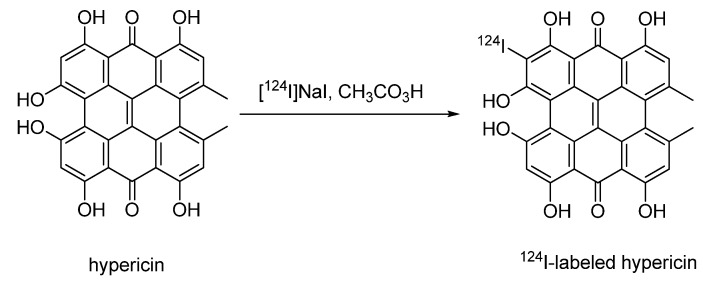
Synthesis of ^124^I-labeled hypericin.

Hypericin as a labeling precursor was synthesised in 65% yield. Conversion of hypercin with sodium iodide in presence of peracetic acid afforded the non-radioactive reference compound. Iodination of hypercin gave various products which could be separated by semi-preparative HPLC. 

Radiosynthesis was performed in the presence of phosphoric acid and peracetic acid as an oxidising agent according to Bormans *et al.*, [21]. [^124^I]NaI in NaOH was slowly added to a 0.5 mg/mL solution of hypericin in EtOH containing 0.5 M H_3_PO_4_ (25 µL) and 0.2 M peracetic acid (50 µL) (Scheme 6). After incubation at room temperature for 30 min, the reaction mixture was purified by HPLC. No information on radiochemical yield, purity or specific activity was given. Cellular uptake studies were performed using ^124^I-labeled hypericin. ^124^I-labeled hypericin provided good quality small animal PET images using PK-C overexpressing malignant glioma mouse xenografts.

#### 4.2.1.3. 2'-Fluoro-2'-deoxy-1-β-D-arabinofuranosyl-5-[^124^I]iodouracil ([^124^I]FIAU)

An ^124^I-labeled uracil derivative [2'-fluoro-2'-deoxy-1-β-D-arabinofuranosyl-5-iodouracil (FIAU)] has been reported as a PET radiotracer for non-invasive imaging of herpes virus thymidine kinase (HSV1-tk) gene transfer and expression for monitoring clinical gene therapy [80].

**Scheme 7:**
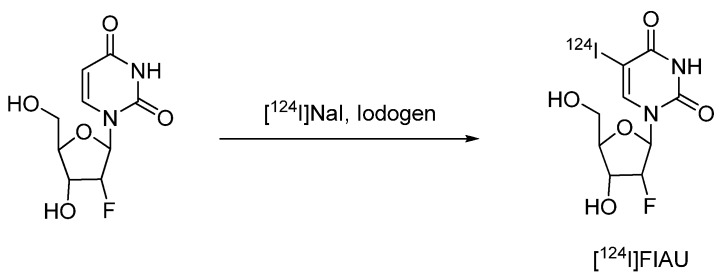
Synthesis of [^124^I]FIAU.

The first synthesis of [^124^I]FIAU was published by Tjuvajev *et al. *in 1996 [80]. [^124^I]FIAU was prepared by hydrogen-to-iodine exchange using 2'-fluoro-2'-deoxy-1-β-D-arabinofuranosyl uracil (FAU) as a labeling precursor and Iodogen^®^ as an oxidising agent. 

An improved synthesis of [^124^I]FIAU was described by Doubrovin *et al.* [81]. This no-carrier-added synthesis of [^124^I]FIAU is based on the reaction of the stannylated labeling precursor 2'-fluoro-2'-deoxy-1-β-D-arabinofuranosyl-5-(tri-*n*-butyltin)-uracil (FTBSnAU) with [^124^I]NaI in the presence of acetic acid and 30% hydrogen peroxide as an oxidising agent. After quenching the reaction by the addition of sodium metabisulfite, [^124^I]FIAU was isolated by solid phase extraction on a C-18 Sep-Pak cartridge. After elution with methanol the product was reconstituted in sterile and pyrogen-free physiological saline (with 5% ethanol added) and passed through a sterile filter. The radiochemical purity was determined to be greater than 97%. The specific activity was not determined. Brust *et al.*, published a slightly modified method based on 5-trimethylstannyl-1-(2-deoxy-2-fluoro-D-arabino-furanosyl) uracil (FTAU) as the labeling precursor. The obtained radiochemical yield was 70%, and the radiochemical purity exceeded 98%. [82]

Small animal PET-studies showed clear accumulation of [^124^I]FIAU in HSV1-tk expressing tumors. In 1998 Tjuvajev *et al.* showed that a retroviral HSV1-tk gene transfer results in an expression level that can be measured by PET. PET imaging at later time points (24 h) is advantageous [83]. Compared with the ^18^F-labeled PET tracer 9-[(3-[^18^F]fluoro-1-hydroxy-2-propoxy)methyl]guanidine ([^18^F]FHPG), [^124^I]FIAU showed significantly higher accumulation in HSV1-tk expressing cells [82]. 

#### 4.2.2. Electrophilic radioiodo-destannylation reactions

#### 4.2.2.1. ^124^I-labeled m-iodophenylpyrrolomorphinan (m-[^124^I]IPPM)

Reports of pharmacological studies on delta-opioid receptors to assess functional receptor expression and advance opioid receptor targeting drugs suggest the use of ^124^I-labeled compound m-iodophenylpyrrolomorphinan (m-[^124^I]IPPM) as a radiotracer for molecular imaging with PET [84]. 

**Scheme 8:**
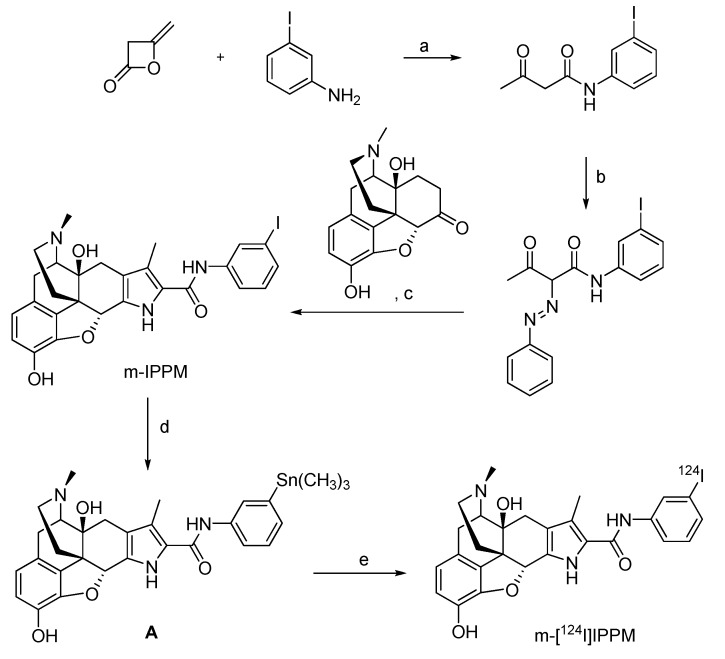
Synthesis of the reference compound m-IPPM and m-[^124^I]IPPM: a) benzene, 0 ºC; b) phenylhydroazonium chloride, 0 ºC, pyridine, room temperature; c) Zn, HOAc/NaOAc, 60 ºC, then reflux; d) Sn_2_Me_6_, PdCl_2_(PPh_3_)_2_; e) [^124^I]NaI, Iodogen^®^ beads.

The synthesis of morphine compounds as reference compound and precursors for radiolabeling with ^124^I is depicted in Scheme 8.

The reference substance m-IPPM was converted into the stannylated labeling precursor **A** by a Pd-mediated reaction with hexamethyldistannane. The radiolabeling was performed via an electrophilic iodo-destannylation reaction using [^124^I]NaI in 5% acetic acid in methanol. Iodogen^®^ beads were used as an oxidising agent to afford m-[^124^I]IPPM. The overall radiochemical yield was 24.5 ± 1.9% (n = 6), and the product was obtained in >98% radiochemical purity at a specific activity of 92.5 ± 44.4 MBq/µmol.

#### 4.2.2.2. [4-(3-[^124^I]iodoanilino)-quinazolin-6-yl]amide-(3-morpholino-4-ylpropyl)amide (mor-pholino-[^124^I]IPQA)

**Scheme 9:**
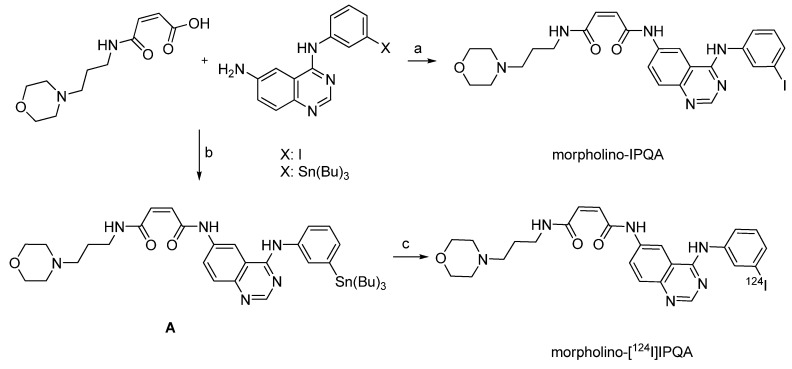
Synthesis of the reference compound morpholino-IPQA, precursor A and radiolabeling: a) EDC, HCl, DMAP, DMF b) EDC, HCl, HOBt, CH_2_Cl_2_ c) [^124^I]NaI, NaOH, MeOH, H_2_O_2_/HOAc (1:3), 1 min vortex, 2 min room temperature.

Molecular imaging of EGFR kinase activity in tumors has been performed with (*E*)-but-2-enedioic acid [4-(3-[^124^I]iodoanilino)-quinazolin-6-yl]amide-(3-morpholino-4-ylpropyl)amide (morpholino-[^124^I]IPQA) [85]. Morpholino-[^124^I]IPQA should allow non-invasive and repetitive imaging of EGFR signalling inhibitors before and during therapy with EGFR inhibitors. Several carbon-11 and fluorine-18 labeled radiotracers for imaging EGFR been developed. However, the short half-lives of these tracers have limited their use in PET imaging [86,87]. On the other hand, the longer half-life of ^124^I should allow imaging at later time points to provide a higher signal-to-noise ratio. Scheme 9 displays the synthesis of the reference and precursor compounds, and the radiolabeling with ^124^I.

As outlined in Scheme 9, radiolabeling was achieved through electrophilic aromatic iodination using stannylated compound **A** and [^124^I]NaI in methanol. Hydrogen peroxide/acetic acid was used as oxidising agent. The reaction mixture was vortexed for one minute and then allowed to stand for two minutes. Afterwards aqueous saturated sodium metabisulfate (0.1 mL) was added to quench the reaction. The product was purified by gradient elution from a C-18 Sep-Pak cartridge. Morpholino-[^124^I]IPQA derivative was obtained in 14.4% radiochemical yield. The radiochemical purity was 85.9%. Preliminary *in vitro *studies using human epidermoid carcinoma (A431), human glioma (U87MG), and human chronic myeloid leukemia (K562) cell lines were conducted with ^131^I-labeled morpholino-IPQA. Small animal PET studies with morpholino-[^124^I]IPQA in rats revealed a significant difference in radiotracer accumulation in EGFR positive A431 tumors and EGFR negative K562 xenografts. In A431 tumor xenografts, tumor-to-muscle and tumor-to-blood concentration ratios of 4.5 and 3.7 were achieved 60 min p.i.. In contrast, tumor-to-muscle and tumor-to-blood concentration ratios were 1.25 and 1.04, respectively, in K562 tumor xenografts [85].

#### 4.2.2.3. ^124^I-labeled 6-anilino-quinazoline derivatives as EGFR inhibitors

**Scheme 10:**
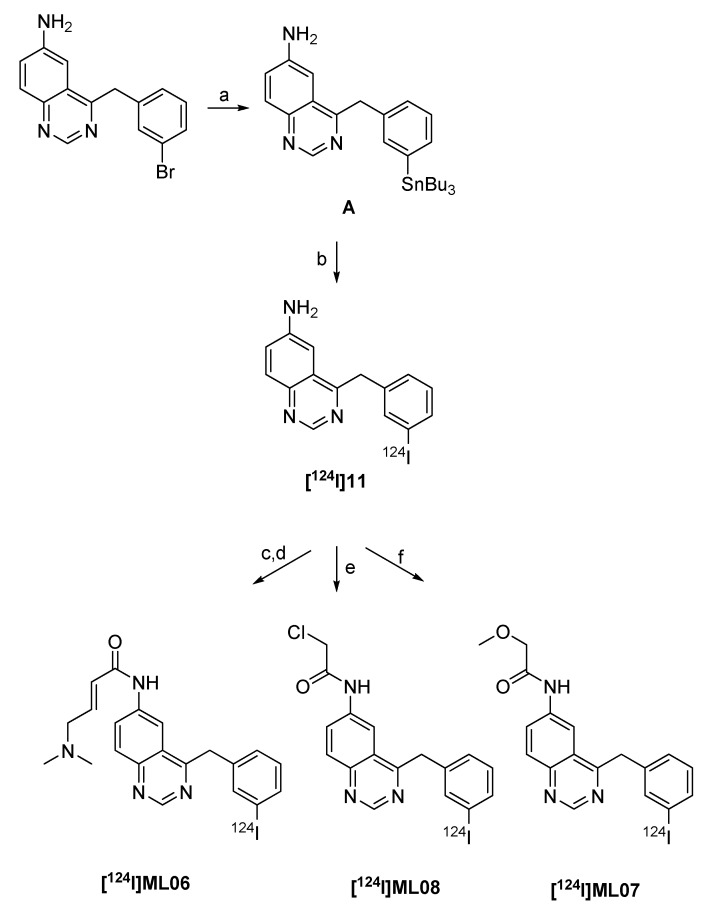
Synthesis of ^124^I-labeled EGFR-inhibitors: a) Sn_2_Bu_6_, Pd(PPh_3_)_4_, THF; b) [^124^I]NaI, NaOH, HCl, Chloramin T; c) BrCH_2_CHCHCOCl; d) Me_2_NH; e) ClCH_2_COCl; f) MeOCH_2_COCl.

Shaul *et al. *also described different ^124^I-labeled radiotracers with irreversible binding to EGFR [88]. The compounds contain a quinazoline backbone. α*-*Methoxyacetamide [^124^I]ML07 was found to be less potent while α-chloroacetamide [^124^I]ML08 and 4-dimethylaminobut-2-enoic amide derivative [^124^I]ML06 possess high potency. The radiolabeling of all compounds is based on iodo-destannylation reaction, as depicted in Scheme 10.

Labeling precursor **A** was treated with [^124^I]NaI in ethanol containing 0.1 N HCl with 1 mg/mL of Chloramine T as the oxidizing agent at room temperature to afford 6-amino-4-[(3-[^124^I]-iodophenyl)amino]quinazoline ([^124^I]11) in 50% radiochemical yield after 15 minutes. The product was purified by solid phase extraction. Compounds [^124^I]ML07 and [^124^I]ML08 were prepared by adding methoxy- or chloroacetylchloride in dry THF to the eluate containing [^124^I]11 at 0 ºC. [^124^I]ML07 was obtained in 28% at a specific activity >222.0 MBq/µmol. [^124^I]ML08 was isolated in 36% at comparable specific activity. Synthesis of [^124^I]ML06 includes three-steps starting from stannyl precursor **A** to provide [^124^I]ML06 in 45% radiochemical yield [88].

#### 4.2.2.4. Synthesis of ^124^I-labeled purpurinimide derivatives

**Scheme 11:**
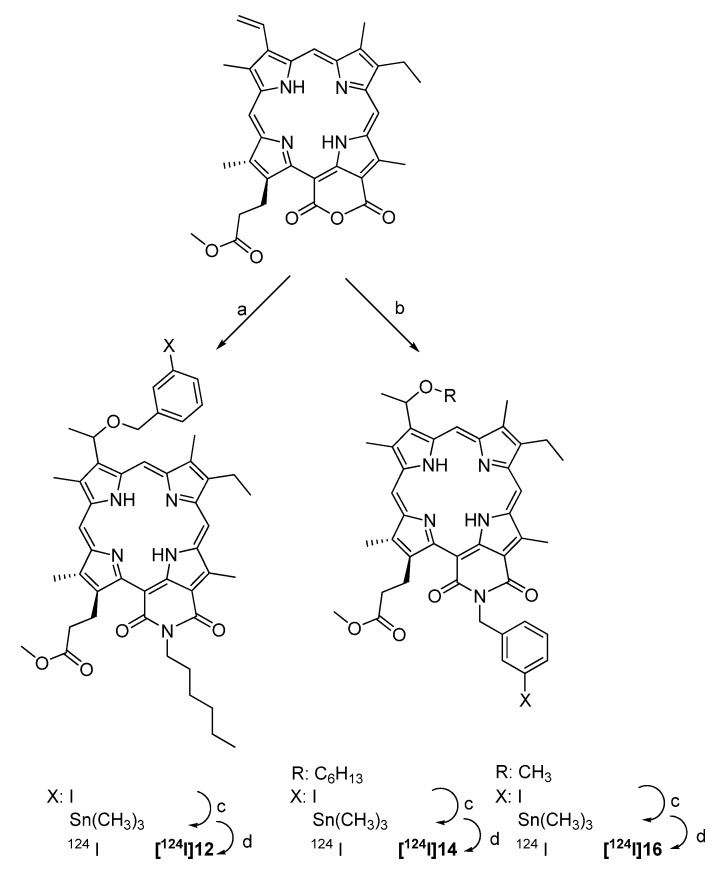
Synthesis of the ^124^I-labeled purpurinimide derivatives [^124^I]12, [^124^I]14 and [^124^I]16: a) (i) *n*-hexyl amine (ii) HBr/AcOH (iii) 3-Iodobenzyl alcohol; b) (i) 3-iodobenzyl amine (ii) HBr/AcOH (iii) R-OH; c) Sn_2_Me_6_, PdCl_2_(PPh_3_)_2_; d) Iodogen^®^, [^124^I]NaI.

Three ^124^I-labeled purpurinimides containing a iodobenzyl substituent have been synthesized and studied as tumor imaging agents with PET [89]. The compounds are porphyrine-based derivatives from methylpheophorbide-*a* isolated from *Spirulina pacifica. *The labeling precursors were prepared by Pd-mediated conversion of the corresponding iodine compounds into trimethyl-stannyl compounds. Radiolabeling was achieved through radioiodo-destannylation in the presence of [^124^I]NaI and Iodogen^®^ beads as an oxidising agent. The reaction took 15 min at room temperature. The final products were purified using HPLC. The radiochemical yield was about 40%, and the specific activity was >37.0 GBq/µmol. The synthesis is given in Scheme 11.

Biological studies using PET were performed in C3H mice bearing RIF tumors. Compounds [^124^I]12 and [^124^I]14 showed exceptionally high liver (32-fold and 8-fold, respectively) and spleen uptake (85-fold and 3-fold, respectively) at 24 h p.i. preventing visualization of the tumor. Compound [^124^I]16 bearing a methyl ester instead of a hexyl ester showed reduced uptake in spleen and liver leading to visible tumor uptake after 96 h p.i.. 

#### 4.2.2.5. Synthesis of ^124^I-labeled 2-(4-iodophenylamino) pyrido[2,3-d]pyrimidin-7-one

Pyrido[2,3-*d*]pyrimidin-7-ones are potent tyrosine kinase inhibitors. A ^124^I-labeled derivative (2-(4-[^124^I]iodophenylamino) pyrido[2,3-*d*]pyrimidin-7-one ([^124^I]18) was prepared as a radiotracer for imaging Abl kinase [90]. 4-Trimethylstannyl- and 4-tri-*N*-butylstannyl-pyridopyrimidinone were prepared as labelling precursors for subsequent radioiodo-destannylation with [^124^I]NaI in the presence of 30% H_2_O_2_/HOAc (1:3) as an oxidizing agent. The product was obtained in 79–86% radiochemical yield with a high radiochemical purity of 99% within 30 min. The synthesis is summarized in Scheme 12. 

**Scheme 12:**
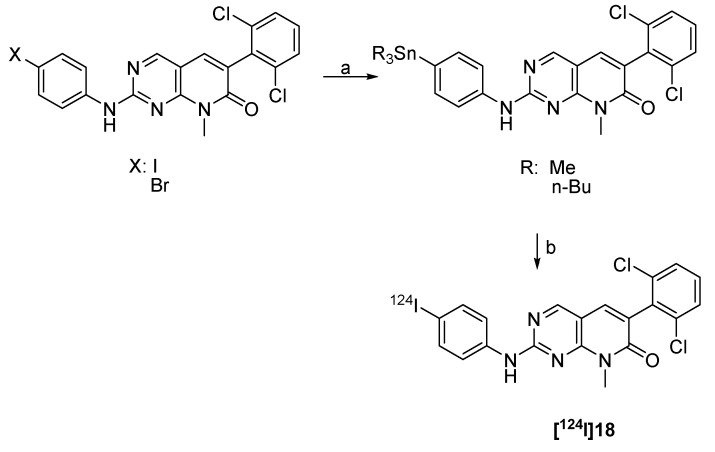
Synthesis of [^124^I]18: a) Sn_2_R_6_, Pd(PPh_3_)_4_, microwave; b) [^124^I]NaI, 30% H_2_O_2_:HOAc (1:3), 10 min, rt.

Radiopharmacological evaluation was reported for the corresponding ^131^I-labeled compound. Cell uptake studies in K562 cells revealed a growth inhibition (IC_50_) value of 2.0 nM and a preferential accumulation in K562 cells compared with the control A431 cells. 

#### 4.2.2.6. Synthesis of n-(morpholin-4-yl)-1-(2,4-dichlorophenyl)-5-(4-[^124^I]iodophenyl)-4-methyl-1h-pyrazole-3-carboxamide ([^124^I]AM281)

**Figure 1:**
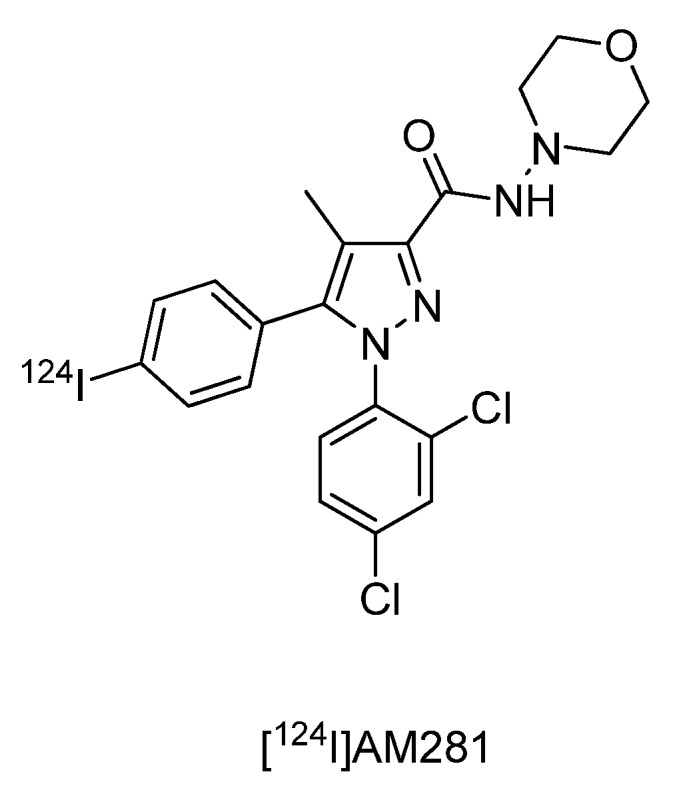
Central cannabinoid CB_1_ receptor ligand[^124^I]AM281.

Studies on central cannabinoid CB_1_ receptors in schizophrenic patients led to the development of ^124^I-labeled imaging probe [^124^I]AM281 (Figure 1) [91]. ^124^I-labeled AM281 was prepared according to a radioiodo-destannylation reaction starting from the corresponding tributyl-stannyl precursor with Chloramine T as an oxidising agent. [^124^I]AM281 was purified via HPLC. 200 MBq [^124^I]NaI was converted into 50 MBq of [^124^I]AM281 for *in vivo* imaging. The PET study revealed an asymmetric CB_1_ receptor binding in basal ganglia. The improved spatial resolution of [^124^I]AM281 as PET radiotracer when compared with [^123^I]AM281 as SPECT radiotracer is compromised by the longer half-life of iodine-124 leading to elevated radiation exposure limiting the amount of applicable activity [91].

#### 4.2.2.7. Synthesis of ^124^I-labeled CDK4/6 inhibitors

Two ^124^I-labeled cyclin-dependent kinase 4/6 inhibitors were developed to study the role of Cdk4/6 during cell proliferation in tumor cells [92]. 80% of human tumors show a deregulation of the cell cycle relevant Cdk4-cyclin D1/retinoblastoma (pRb)/E2F signal cascade resulting in uncontrolled tumor growth. Radiolabeled Cdk4 inhibitors have been suggested as promising molecular probes for imaging tumor cell proliferation. Based on selective Cdk4/6 inhibitors reported in the literature, two compounds were selected for radiolabeling via radioiodo-destannylation with iodine-124 [93,94]. The synthesis is given in Scheme 12.

**Scheme 12:**
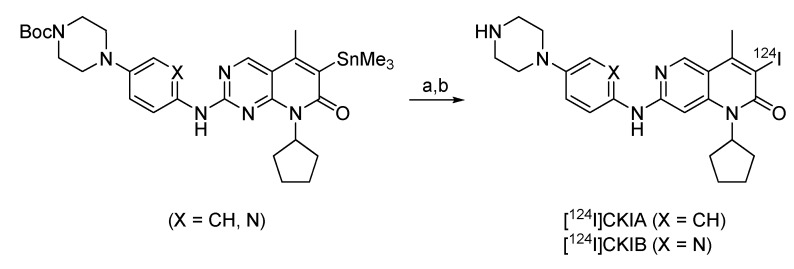
Radiolabeling of the Cdk4/6 inhibitors: a) [^124^I]NaI, Iodogen^®^, 5% HOAc in MeOH, DMSO, room temperature, 10 min; b) TFA.

*C*-6 Trimethylstannyl-substituted compounds were used as labeling precursors. Regioselective incorporation of I-124 into the *C*-6 position of pyrido[2,3-*d*]-pyrimidine compounds was accomplished with [^124^I]NaI in the presence of mild oxidizing agents such as immobilised Chloramine-T (Iodobeads^®^) or Iodogen^®^ followed by removal of the Boc protecting group to afford the desired radiolabeled compounds [^124^I]CKIA and [^124^I]CKIB. The optimisation was performed with trimethylstannyl-precursor (X = CH) and 2-3 MBq of [^124^I]NaI (3–4 µL of [^124^I]NaI in 0.25 M NaOH) at room temperature. The results clearly demonstrate that using 100–200 µg of labeling precursor (150 µL 1:3 mixture of DMSO and 5% acetic acid in methanol) and 1 Iodobead® affords 90% of radiolabelled compound Boc-[124]CKIA after 10 min with no side products. In a larger scale radiosynthesis (up to 275 MBq of [^124^I]NaI) the use of iodobeads as the oxidising agent caused unexpected problems as most of the radioactivity was irreversibly attached to the iodobeads. When using Iodogen^®^-coated tubes no undesired loss of activity was observed. Deprotection was performed with trifluoroacetic acid (TFA) at 50 ºC in 20 min to give the desired compound [^124^I]CKIA in radiochemical yields of 45–65% The product was purified using semi-preparative HPLC column in combination with SPE on a Waters Sep-Pak-tC-18 cartridge. After HPLC the product was loaded onto the cartridge, washed with water and finally eluted with 1 ml ethanol to afford compound [^124^I]CKIA with a radiochemical yield of 33.6%. Optimised reaction conditions were also applied to the synthesis of compound [^124^I]CKIB, which was prepared with a radiochemical yield of 28%. Both compounds could be synthesised in high radiochemical purity (>95%) with a specific activites suitable for radiopharmaceutical investigations (35 GBq/µmol for [^124^I]CKIA and 25 GBq/µmol for [^124^I]CKIB). The log D (pH 7.4) values for compounds [^124^I]CKIA and [^124^I]CKIB were 2.77 ± 0.13 and 1.99 ± 0.03, respectively [92]. Graf *et al.,* showed that the reference substances, when applied in nM-µM-scale were able to induce cell cycle arrest in G_1_-Phase in HT-29 and FaDu cell lines [95] after 24 h. Also a high *in vitro *radiotracer cell uptake in HT-29 and FaDu tumor cells (approximately 750–850 %ID/mg protein [^124^I]CKIA and 900–1,000 %ID/mg protein [^124^I]CKIB (means) after 1 h) at 37 ºC was measured. Initial small animal PET imaging was performed in FaDu xenograft bearing mice revealing a predominantly hepatobiliary elimination, with no specific radioactivity accumulation in other organs and tissues, including the tumour. Biodistribution profile and *ex vivo* autoradiography-studies confirmed a clearance of the activity from most organs and tissues within 60 min after radiotracer administration, except for thyroid. The observed increase of radioactivity concentration in the thyroid is indicative of *in vivo* radio-deiodination and accumulation of free [^124^I]iodide in the thyroid over time [92,95].

### 4.3. Radioiodination of peptides and proteins

#### 4.3.1. Direct labeling of peptides and proteins with ^124^I

Methods for radioiodination of peptides and proteins should be mild and rapid while providing high radiochemical yields. 

^124^I-labeled human insulin was prepared by Glaser *et al.* in 2001 by direct electrophilic iodination at the A_14_-tyrosine residue [96]. Radioiodinated insulin labelled at A_14_-tyrosine residue is known to retain receptor binding properties and biological activity as the native hormone. 

Different oxidising agents, such as Iodogen^®^, Chloramine-T, *N*-bromosuccinimide, and lactoperoxidase-hydrogen peroxide were investigated for direct radiolabeling of insulin with iodine-124 affording comparable radiochemical yields of about 33%.

Radiolabeling was performed in sodium phosphate buffer (pH 7.6) within 30 seconds. The specific radioactivity after single HPLC was 12.5 GBq/µmol and 25.8 GBq/µmol after tandem-HPLC. PET and biodistribution studies using ^125^I- and ^124^I-labeled insulin in rats showed uptake in both the myocardium and the liver. This uptake could be blocked with unlabeled insulin. Studies on the *in vivo* stability of radiolabeled insulin demonstrated rapid metabolism [96,97]. 

The long physical half-life of iodine-124 (t_1/2_ = 4.2 d) is particularly well suited for labelling large molecules like antibodies. The relatively long half-life allows PET imaging at late time points (>24 h) ensuring sufficient accumulation of the radiolabeled antibody in the target tissue (e.g. tumor). Various ^124^I-labeled antibodies have been used for molecular imaging and therapy breast cancer, colorectal cancer, ovarian cancer and neuroblastoma. 

The radiolabeling was accomplished via direct radioiodination at tyrosyl and, to a lesser extent, histidyl residues of the antibody and protein using different oxidizing agents such as Iodogen^®^, Chloramin T (CAT) and *N*-bromosuccinimide (NBS) in buffer at pH below 8.

Table 2 summarizes ^124^I-labeled antibodies and proteins which have been prepared via direct electrophilic radioiodination. 

**Table 2:** Direct labeling of proteins and antibodies with ^124^I.

Method	Imaging target	Antibody	Results	Reference
Iodogen^®^	HER2	Anti-HER2	RCY: 13.8–18.8%;	[98]
			A_S_: 33-102 MBq/mg,	
	positive tumours	Diabody	RCP: >93.5%	
	Apoptotic cells	Annexin V	RCY: 20–70%	[99,100]
		Annexin V	RCY: 6–12%	[101,102,103]
			RCP: 95%	
		MBP-Annexin V		[104]
	CD44v6	cMAb U36	RCY: 72%	[105,106]
	positive cells		RCP: >97%	
			A_S_: 32.6 MBq/mg	
	squamous cell			
	carcinoma			
	CEA	Anti-CEA	RCY: 33–88%	[107]
		mini- and diabodies		
CAT-method	Apoptotic cells	Annexin V	RCY: 4-6%,	[103]
			RCP: >99%,	
			A_S_: 8.6 GBq/µmol	
	Human glioma	3F8 mAb	RCY: >90%	[108,109,110]
		A33 mAb	RCY: 55%	[110]
	Colon cancer	huA33 mAb	RCY: 40%,	[111]
			RCP >98%,	
			A_S_: 7.4 MBq/mg	
	CEA	mAb CE-25,	RCY: 70–80%,	[112]
		CE 4-8-13		
	Ovarian cancer	MX35 or MH99	RCY: >87%	[113]
NBS-method	Breast cancer	C-erbB2	RCY: 96%	[114]
		HMFG1		[115]

RCY = radiochemical yield; RCP = radiochemical purity; A_S_ = specific activity.

#### 4.3.2. ^124^I-labeled prosthetic groups for radiolabeling of peptides and proteins

Various prosthetic groups have been developed for radiolabeling peptides and proteins with radioiodine, including ^124^I. Prominent examples are the Bolton-Hunter reagent *N*-succinimidyl 3-(4-hydroxy-5-[^124^I]iodophenyl) propionate ([^124^I]I-SHPP,) succinimidyl-3-[^124^I]iodobenzoate (3-[^124^I]SIB) and succinimidyl-4-[^124^I]iodobenzoate (4-[^124^I]SIB). 

**Scheme 13:**
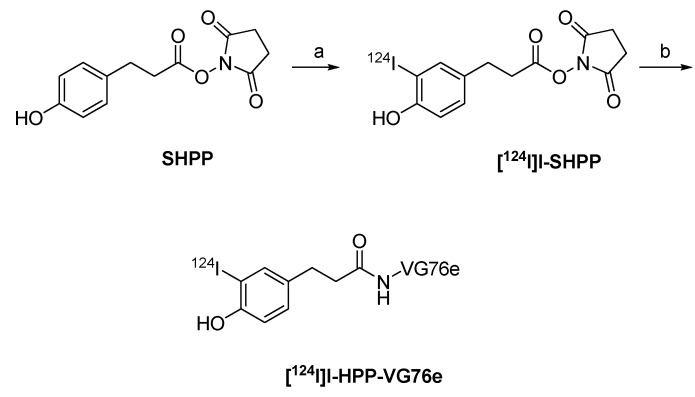
Synthesis of [^124^I]SHPP and radiolabeling of VG76e: a) [^124^I]NaI, Iodogen^®^ (pH 6.5); b) VG76e, pH 8.5, 0 ºC.

^124^I-labeled Bolton-Hunter reagent [^124^I]I-SHPP was used for labelling an antibody VG76e targeting VEGFR to monitor therapy that relies on the VEGF pathway (Scheme 13) [116].

The Bolton-Hunter reagent was first introduced by Bolton and Hunter in 1972 to copy iodo-tyrosyl residues as observed for the direct labeling of tyrosyl residues with radio-iodine. The Bolton-Hunter reagent reacts with lysine residues of peptides or proteins. It is more stable towards radio-deiodination. 

Synthesis of [^124^I]I-SHPP was carried out by incubating SHPP with a mixture of [^124^I]NaI in HCl and sodium phosphate buffer (pH 6.5) in a Reacti-vial™ coated with Iodogen^®^. After 1 min the solution was transferred into a second Reacti-vial™ containing anhydrous benzene-DMF (40:1) to quench the reaction. The organic layer was removed and dried by passing through a Pasteur pipette containing sodium sulphate. The sodium sulphate was washed with benzene to extract the additional product. The radiochemical yield of [^124^I]I-SHPP was 25–58%.

The VG76e was diluted in sodium borate buffer (pH 8.5) and incubated with [^124^I]I-SHPP for 1 h at 0 ºC. Radiolabeled antibody was purified by gel filtration. The average radiochemical yield of [^124^I]I-SHPP-VG76e was 16.3 ± 8.7% with an specific activity of 11.1 GBq/µmol. PET images of HT1080-26.6 tumor-bearing mice clearly showed accumulation of the radiotracer in the tumor. The best contrast was achieved 24 h p.i. [116,117].

[^124^I]I-SHPP was used for the radiolabeling of a anti-HER2 diabody for PET imaging of HER2-positive tumors [98]. The diabody was labeled directly according to the Iodogen^®^ method and indirectly using Bolton-Hunter reagent [^124^I]I-SHPP. Direct radioiodination afforded the ^124^I-labeled diabody in radiochemical yields of 14–19% at a specific activity of 33–102 MBq/mg. Indirect radiolabeling by means of [^124^I]I-SHPP yield resulted in radiochemical yields of 6–8%. The specific activity was 37–46 MBq/mg.

*In vivo* studies revealed a higher tendency of radio-deiodination and subsequent radioactivity accumulation in the thyroid and stomach in the case of directly radioiodinated diabody. However, the metabolically more stable Bolton-Hunter conjugated diabody showed reduced immunoreactivity compared to the diabody prepared via direct radioiodination. The reduced immunoreactivity did not prevent PET imaging of HER2-expressing tumors [98].

JAA-F11 antibody used to localise Thomsen-Friedenreich alpha-linked antigen (TF-Ag)* in vivo* was labeled with [^124^I]I-SHPP for imaging of breast cancer metastases [118]. The antibody was also labeled according to the Iodogen^®^ method to afford the radiolabeled antibody in radiochemical yields of 20%. The directly radiolabeled antibody showed diminished immunoactivity compared with the antibody labeled by means of a prosthetic group ([^124^I]I-SHPP). Indirect labeling using Bolton-Hunter reagent [^124^I]I-SHPP was achieved in radiochemical yields of 30%. The specific activity was determined to be 57 MBq/mg. Biodistribution studies showed uptake of the radiotracer in TF-AG positive tumors [118].

**Figure 2:**
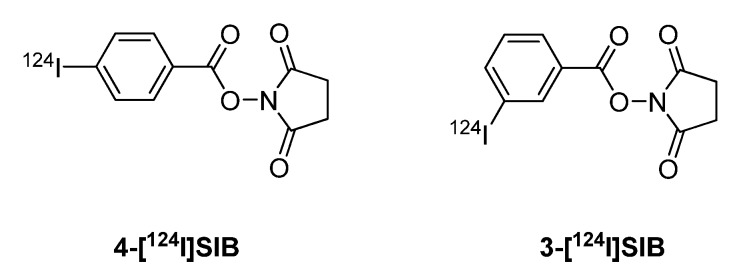
Structures of 4-[^124^I]SIB and 3-[^124^I]SIB.

Various antibodies were labeled using the prosthetic group [^124^I]SIB. Synthesis of the prosthetic groups 3-[^124^I]SIB and 4-[^124^I]SIB was reported by Koziorowski *et al.* (Figure 2) [119]. This synthesis is based on a radio-destannylation reaction starting from the corresponding trimethylstannyl precursor using Iodogen^®^ or iodobeads as the oxidizing agent. 

Collingridge *et al. *[101] and Glaser *et al.* [102] published iodine-124 labelling of annexin V using m-[^124^I]SIB as a prosthetic group. Annexin V, a 36 kDa protein, is used to measure apoptosis in histopathology. An increasing number of reports also deal with radiolabeled annexin V as molecular probe for imaging apoptosis *in vivo*. Annexin V has been labeled with various radioiodine isotopes like iodine-125, -131 and -123. Radiolabeling with iodine-124 provides radiolabeled annexin V suitable for PET imaging. 

**Scheme 14:**
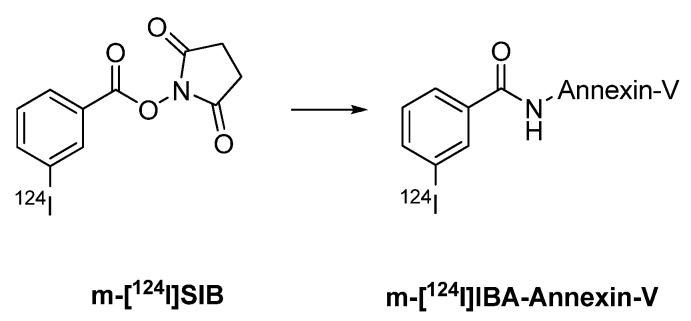
Labeling of Annexin V with 3-[^124^I]SIB.

Direct labeling of annexin V with [^124^I]NaI and chloramine T as the oxidizing agent gave ^124^I-labeled annexin V in radiochemical yields of 22.3 ± 2.6%. Indirect labeling of annexin V involved coupling with 3-[^124^I]SIB, and the radiochemical yield of ^124^I-labeled annexin V was 34% (Scheme 14) [101,102,103].

**Scheme 15:**
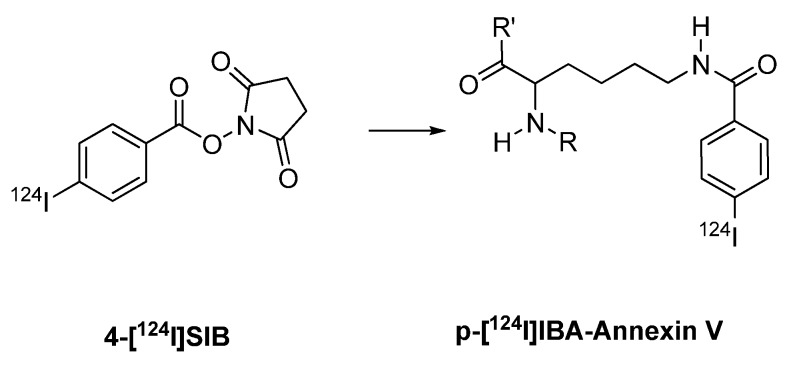
Labeling of a peptide/protein with 4-[^124^I]SIB.

Prosthetic group 4-[^124^I]SIB was also used for the radiolabeling of annexin V (Scheme 15) [104]. The radiochemical yield was 20–30%. 

The indirectly radiolabeled annexin V (with 3-[^124^I]SIB or 4-[^124^I]SIB) showed higher *in vivo* stability when compared with the directly labelled annexin V. However, conjugation of annexin V with 3-[^124^I]SIB or 4-[^124^I]SIB resulted in an increased radioactivity accumulation in the kidneys. Therefore, directly labeled ^124^I annexin V seems to be more suitable for PET imaging of apoptosis *in vivo* [104].

## 5. Summary and Conclusions

The present review has summarized the application of ^124^I as a PET radionuclide for molecular imaging. Over the last four decades, radioiodinated radiopharmaceuticals have played an important role in nuclear medicine. The rapidly growing field of molecular imaging has stimulated research on novel positron-emitting radionuclides, especially with longer half-lives. The positron-emitting radiohalogen ^124^I with its 4.2 d half-life is particularly attractive for *in vivo* detection and quantification of longer term biological and physiological processes. The long half-life of ^124^I is especially suited for *in vivo* studies of the prolonged time course of uptake of higher molecular weight compounds like monoclonal antibodies (MAbs) in solid tumors. Moreover, the long half-life allows serial scanning protocols over a period of several days. 

Recent advances in radionuclide production (targetry and target processing) allow the convenient production of sufficient quantities of ^124^I on small biomedical cyclotrons for molecular imaging purposes. Radioiodination chemistry with ^124^I relies on established radioiodine labeling methods, which can be subdivided into nucleophilic and electrophilic substitution reactions. The ease of oxidizing iodide into electropositive iodine species make electrophilic labeling methods the most frequently employed radioiodination technique. Over the last decade, numerous small molecules and larger compounds like proteins and antibodies have been successfully labeled with ^124^I. 

The future advances in probe development will lead to the development of novel innovative radiopharmaceuticals for specific molecular targeting for both imaging and therapy. The favorable 4.2 d half-life, the ease of production on widely available small medical cyclotrons, and the well-established radioiodine radiochemistry will make ^124^I an important radiohalogen for future radiotracer development in the rapidly growing field of molecular imaging. 
